# Evidence that Xrn1 is in complex with Gcn1, and is required for full levels of eIF2α phosphorylation

**DOI:** 10.1042/BCJ20220531

**Published:** 2024-03-28

**Authors:** Renuka Shanmugam, Reuben Anderson, Anja H. Schiemann, Evelyn Sattlegger

**Affiliations:** 1 School of Natural Sciences, Massey University, Auckland, New Zealand; 2 School of Natural Sciences, Massey University, Palmerston North, New Zealand; 3 Maurice Wilkins Centre for Molecular BioDiscovery, Massey University, Palmerston North, New Zealand

**Keywords:** Gcn1, Gcn2, molecular biology, *Sacharomyces cerevisiae*, starvation signaling, Xrn1

## Abstract

The protein kinase Gcn2 and its effector protein Gcn1 are part of the general amino acid control signalling (GAAC) pathway best known in yeast for its function in maintaining amino acid homeostasis. Under amino acid limitation, Gcn2 becomes activated, subsequently increasing the levels of phosphorylated eIF2α (eIF2α-P). This leads to the increased translation of transcriptional regulators, such as Gcn4 in yeast and ATF4 in mammals, and subsequent re-programming of the cell's gene transcription profile, thereby allowing cells to cope with starvation. Xrn1 is involved in RNA decay, quality control and processing. We found that Xrn1 co-precipitates Gcn1 and Gcn2, suggesting that these three proteins are in the same complex. Growth under starvation conditions was dependent on Xrn1 but not on Xrn1-ribosome association, and this correlated with reduced eIF2α-P levels. Constitutively active Gcn2 leads to a growth defect due to eIF2α-hyperphosphorylation, and we found that this phenotype was independent of Xrn1, suggesting that *xrn1* deletion does not enhance eIF2α de-phosphorylation. Our study provides evidence that Xrn1 is required for efficient Gcn2 activation, directly or indirectly. Thus, we have uncovered a potential new link between RNA metabolism and the GAAC.

## Introduction

Virtually all Eukaryotic cells harbour an ancient signal transduction pathway that allows them to cope with amino acid starvation conditions [1]. In this pathway, the cytosolic protein kinase Gcn2 monitors amino acid availability. Under amino acid limitation, Gcn2 phosphorylates the alpha subunit of translation initiation factor 2 (eIF2α).

eIF2 in its GTP-bound form binds initiator methionyl-tRNA^Met^ (Met-tRNA_i_^Met^) to form the ternary complex that delivers the Met-tRNA_i_^Met^ to the ribosome during translation initiation [2]. Once the translation start codon has been detected, eIF2 is released in its GDP-bound form. eIF2 needs to be recycled to its eIF2-GTP-bound form by its guanine nucleotide exchange factor (GEF) eIF2B, to be able to form the next ternary complex. eIF2 phosphorylation converts eIF2 from a substrate to an inhibitor of eIF2B, thereby leading to reduced cellular levels of ternary complex. As a consequence, protein synthesis is affected in two ways, reduction in global protein synthesis, and at the same time increased translation of specific mRNAs coding for transcription factors, such as Gcn4 in yeast or ATF4 in mammals [2,3]. The regulation of *GCN4/ATF4* translation is mediated by upstream open reading frames (uORFs) present in the 5′ untranslated region of the mRNA [2]. eIF2α phosphorylation and concomitant reduction in availability of ternary complexes allows ribosomes to overcome the inhibitory function of the uORFs and instead initiate at the main open reading frame. The resulting increased Gcn4/ATF4 protein levels regulate the transcription of many genes, including the increased transcription of genes coding for amino acid biosynthetic enzymes [2,4]. In nature, cells usually do not experience such harsh starvation conditions as those imposed in the laboratory, since they start to already respond to the onset of starvation. This means a more modest level of Gcn2 activation, and a more modest increase in eIF2 phosphorylation. Hence, the resulting increased translation of Gcn4/ATF4 is the most critical starvation response rather than the reduction in global protein synthesis [2,3].

So far, this starvation pathway has been best studied in the yeast *Saccharomyces cerevisiae*. Even when starved for only one amino acid, this pathway induces the expression of enzymes belonging to many amino acid biosynthetic pathways, leading to the *de novo* synthesis of more than just the missing amino acid. For this reason, in yeast this pathway was called general amino acid control (GAAC).

Gcn2 is absolutely dependent on its effector protein Gcn1 for its activation [5], and it must directly bind to Gcn1, via the N-terminal RWD domain (a domain found in RING finger-containing proteins, WD-repeat-containing proteins, and yeast DEAD (DEXD)-like helicases) in Gcn2 and the RWD binding domain (RWDBD) in Gcn1 [6]. The R2259A substitution in the RWDBD of Gcn1 abolishes Gcn2-binding *in vivo* and *in vitro*, and impairs Gcn2 activation *in vivo*, but does not affect any other known Gcn1 functions [6], suggesting that Arg-2259 is a direct Gcn2 contact point. Since in the cell extract of *gcn1Δ* strains Gcn2 is still enzymatically active, this suggests that Gcn1 is not required for the Gcn2 enzymatic activity *per se*, but that Gcn1 is directly involved in transfer of the starvation signal to Gcn2 [5–7]. Gcn1 [7] and Gcn2 [8] each bind to the ribosome, and this interaction is important for full Gcn2 activation. In addition, in Gcn1 as well as Gcn2, the regions required for ribosome binding do not overlap with those required for direct Gcn1-Gcn2 interaction [6]. This suggests that Gcn1, Gcn2 and the ribosome can form a trimeric complex.

The exact mechanism by which Gcn2 detects starvation is still not fully understood. Currently two models were proposed which do not necessarily exclude each other. In the first working model, Gcn2 and Gcn1 form a trimeric complex with the ribosome [5,6]. Under starvation conditions, when the cognate charged tRNA is not available, an uncharged tRNA enters the ribosomal A-site in a codon specific manner. This tRNA is then transferred to the Histidyl-tRNA synthesis-like domain of Gcn2, leading to Gcn2 auto-phosphorylation [2]. Activated Gcn2 then phosphorylates its substate eIF2α. In a second working model, ribosomal stalk proteins are involved in mediating Gcn2 activation [9–11]. Unavailability of a cognate aminoacylated tRNA allows the ribosomal stalk proteins to interact with Gcn2 to mediate the stimulation of its kinase domain [10]. The link between uncharged tRNAs and the P-stalk remains to be determined in view of Gcn2 activation under amino acid starvation in yeast and mammals. No matter the mechanism of Gcn2 activation, yeast studies suggest that direct Gcn1–Gcn2 interaction, and the association of Gcn2 and Gcn1 with the ribosome, are required for Gcn2 activation [6,8,12]. Supporting the idea that the same is true in mammals, it has recently been shown that deletion of Gcn1 in mice abolishes Gcn2 activation [13]. Gcn2 has been found to also play a crucial role in responding to ribotoxic stress elicited by colliding ribosomes [14].

Gcn2 is also implicated in a large array of other biological processes, such as coping with glucose starvation, cell cycle regulation, neuronal development, the immune system, and memory formation [1]. This implies that Gcn2 must be tightly regulated in order to ensure that it executes the correct function at the correct time, cellular location, and organ. Not surprisingly, Gcn2 has been linked to various diseases and disorders, such as cancer and Alzheimer's disease [1,15], highlighting the need to better understand the molecular mechanisms underlying Gcn2 regulation. Curiously, it appears that Gcn1 is required for the various Gcn2 functions unrelated to overcoming amino acid starvation [1], underscoring the importance of Gcn1 for Gcn2 function and regulation.

Gcn1 is a large cytoplasmic protein with a molecular mass of 296 kDa with no known enzymatic activity [5]. Only the Gcn1 middle portion has significant homology to another known protein, which is the N-terminus of the fungal translation elongation factor 3 (eEF3) [5]. Computational analyses suggest that Gcn1 consists almost entirely of HEAT repeats [16], and this was supported by the computational model established with high confidence for the RWDBD of Gcn1 [17], as well as by the cryoEM structure of Gcn1 bound to the ribosome [18]. The abbreviation HEAT was derived from proteins in which the repeats were first identified; Huntington, Elongation factor 3, Protein phosphatase 2A and Target of rapamycin [16]. Proteins containing HEAT repeats are usually large and interact with a wide variety of proteins [16]. It appears that HEAT repeat proteins function as scaffold proteins, forming a platform on which signalling molecules can assemble to form a multiprotein complex, thereby allowing the co-ordination of regulation in a temporal as well as spatial manner [19]. Together, this suggests that Gcn1 functions as a scaffold protein to allow the modulation of Gcn2 activity. In fact, a couple of proteins have already been identified that bind to Gcn1.

The first protein discovered to bind to Gcn1 was Gcn20 [20]. Gcn20 is required, but not essential, for Gcn2 activation [7,20]. Gcn1-ribosome co-sedimentation assays suggest that Gcn20 modulates the affinity of Gcn1 to the ribosome, supporting the idea that Gcn20 fine-tunes Gcn1-ribosome interaction in response to certain non-yet-known conditions, and that way may modulate the level of Gcn1-mediated Gcn2 activation.

Experimental studies revealed that the N-terminal ¾ of Gcn1 (residues 1–2052) is required for ribosome binding, suggesting that Gcn1 contains many weak binding sites that together are strong enough for providing sufficient affinity to the ribosome [6,12]. Supporting this idea, cryo EM studies showed Gcn1 contacting ribosomal disomes almost throughout its entire length [18]. Since disomes result from a translating ribosome rear-ending a stalled ribosome leading to ribotoxic stress [21], this supports the idea that Gcn1 as well as Gcn2 are involved in responding to ribotoxic stress. So far, the small ribosomal protein Rps10 was shown to directly contact Gcn1, and disruption of this interaction reduces the efficiency of Gcn2 activation [22]. Rps10 may be necessary to keep the functional part of Gcn1 in sufficient proximity to the ribosome to promote efficient Gcn2 activation.

The first and so-far best characterised Gcn2 inhibitor, that is also a Gcn1 binding protein, is Yih1 in yeast (Yeast Impact Homologue 1) and the mammalian counterpart called IMPACT (imprinted with ancient domain) [1]. As found for Gcn2, Yih1/IMPACT contains an N-terminal RWD domain that binds to the Gcn1 RWDBD in an Arg-2259 dependent fashion [23,24]. This way, Yih1/IMPACT competes with Gcn2 for Gcn1-binding in yeast as well as mammals [23–25]. As a consequence, Gcn1–Gcn2 interaction is reduced, and so is Gcn2 activation. Yih1 as well as IMPACT are located on the ribosome [26,27], raising the intriguing possibility that Yih1/IMPACT is located in close proximity to Gcn1 and Gcn2 on the ribosome, allowing instant Gcn2 inhibition and reversal of inhibition in a spatiotemporal manner in the cell. Since deletion of *YIH1* does not lead to increased Gcn2 activity, this suggests that Yih1/IMPACT inhibits Gcn2 only under certain circumstances or in certain locations in the cell [23], or specific organs in an organism such as the hypothalamus [28]. The cue that triggers Yih1/IMPACT to inhibit Gcn2 remains to be uncovered. So far it is only known that actin dynamics affects IMPACT's ability to inhibit Gcn2 [25,29]. Gir2 in yeast, or DFRP2 in mammals, also contains an N-terminal RWD domain, and so far for Gir2 it has been shown that it inhibits Gcn2 by binding to Gcn1, as found for Yih1/IMPACT [30]. The role of Gir2 is to dampen the Gcn2 response under prolonged stress conditions [31].

Taken together, evidence is accumulating that Gcn1 is a scaffold protein that binds other proteins to allow adjustment of Gcn2 activity — and thus modulation of the GAAC pathway response — to the cell's needs. We interrogated published large-scale interactome studies [32–35], to identify proteins potentially in complex with Gcn1. Among these proteins, Xrn1 was found to be in the same complex as Gcn1 [33,35]. For that reason, we here aimed to investigate whether Xrn1 is relevant for the functioning of the GAAC pathway. Xrn1 is a 3′ → 5′ exonuclease that is best known for its involvement in mRNA decay and quality control, as well as translational regulation through modifying the abundance of specific mRNA species via miRNA, siRNA, and lncRNA [36]. We found that cells deleted for *XRN1* were less able to grow under starvation conditions, and this correlated with reduced phosphorylation levels of eIF2α. Constitutively active Gcn2 is known to cause slow growth due to eIF2α hyper-phosphorylation, and concomitant impairment of general protein translation [37]. Deletion of *XRN1* did not revert this growth defect, nor did it impair eIF2α hyper-phosphorylation, suggesting that Xrn1 is not required for the Gcn2 enzymatic function *per se*, nor for the recognition of its substrate eIF2α. Furthermore, this suggested that *XRN1* deletion did not simply lead to enhanced rates of eIF2α de-phosphorylation. mEGFP inserted in Xrn1 in-frame after Ser-235 sterically prevents Xrn1-ribosome binding [38], and this Xrn1-mEGFP was still able to complement an *xrn1Δ* strain for growth under starvation conditions, suggesting that Xrn1-ribosome interaction is not critical for the GAAC response. Our co-precipitation studies suggest that Xrn1 is in complex with Gcn1 [33,35], and that Gcn2 is part of this complex as well. Together, our findings suggest that Xrn1 promotes efficient Gcn2 activation, directly or indirectly, and potential mechanisms are laid out in the discussion section.

## Results

### *XRN1* deletion leads to impaired growth under starvation conditions.

Considering that for *in vivo* activation, Gcn2 must directly bind to its effector protein Gcn1 [5], and that in interactome studies Xrn1 was found to be potentially in complex with Gcn1 [33,35], this raised the possibility that Xrn1 contacts Gcn1 to modulate the level of Gcn2 activation. To test this notion, we wanted to investigate whether *XRN1* deletion affects Gcn2 activation *in vivo*. For this, we took advantage of the fact that *in vivo*, Gcn2 activity can be easily scored in semi-quantitative growth assays, where cells are grown in the absence or presence of sulfometuron methyl (SM), a drug causing starvation for branched-chain amino acids [39]. Only cells able to activate Gcn2 can grow in presence of SM. The more Gcn2 activation is hampered, the weaker the growth in presence of SM.

**Figure 1.**
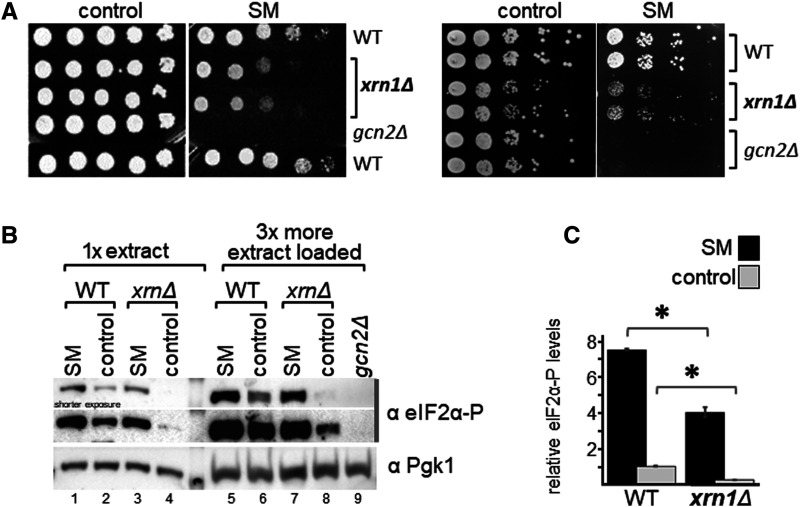
*XRN1* deletion renders cells sensitive to sulfometuron methyl (SM). (**A**) Left panel: The yeast strains deleted for the indicated gene, and the isogenic wild-type strain (WT), were grown to saturation, subjected to 10-fold serial dilutions, and 5 µl of each dilution transferred to solid medium containing 1 μg/ml SM or not (control). Right panel: The same assay was performed, just that cells were grown to exponential phase in liquid medium to an OD of 1, before conducting the semi-quantitative growth assay. (**B**) *XRN1* deletion leads to reduced levels of phosphorylated eIF2α (eIF2α-P). The indicated strains were grown to exponential phase, and then exposed for 1 h to 1 μg/ml SM, or not (control) before harvesting. Whole cell extracts were generated and subjected to SDS–PAGE and westerns using antibodies specific against the phosphorylated form of eIF2α, and Pgk1 as loading control. A representative result is shown. (**C**) Western signals in (**B**) were quantified and the eIF2α-P levels determined relative to that of Pgk1, and plotted in a bar graph relative to the eIF2α-P/Pgk1 ratio of the non-starved wild-type. Error bars depict the standard error, and stars indicate significant differences between values (Student's *t*-test, *P* ≤ 0.05). Quantifications were performed from four biological replicates.

For this growth assay, saturated overnight cultures of wild-type (WT) yeast, and isogenic strains deleted for *XRN1* or *GCN2*, were subjected to 10-fold serial dilutions, and aliquots were transferred to solid medium containing SM or not. As expected, WT yeast was able to grow in presence of SM, but not a *gcn2Δ* strain (Figure 1A, left panel). We found that in presence of SM, the growth of *xrn1Δ* strains was impaired as compared with the WT strain. Given that in these growth assays the cells had to exit stationary phase while already exposed to SM, this raised the possibility that the observed SM sensitivity (SM^s^) phenotype of *xrn1Δ* strains was due to an impaired ability to re-enter the cell cycle, rather than impaired Gcn2 activation. To test this, we repeated the growth assay but transferred exponentially growing cells onto solid medium. We found that even under these conditions the *xrn1Δ* strain exhibited a SM^s^ sensitivity (Figure 1A, right panel), which is in agreement with the idea that Gcn2 activation was hampered in the *xrn1Δ* strain. The fact that in contrast with the *gcn2Δ* strain, the *xrn1Δ* strain was still able to grow to some extent on the SM medium, this suggested that Xrn1 is not essential for Gcn2 activation, but required for full Gcn2 activation.

### *XRN1* deletion leads to reduced levels of eIF2α phosphorylation

Next, we aimed to obtain evidence that the SM^s^ phenotype is due to impaired Gcn2 activation, by scoring for the phosphorylation level of eIF2α (eIF2α-P), the substrate of Gcn2. For this, cells were grown to exponential phase in liquid medium, and exposed for 1 h to 1 µg/ml SM before harvesting. Whole cell extracts were generated and subjected to SDS polyacrylamide electrophoresis (SDS–PAGE), and immunoblotting using antibodies against phosphorylated eIF2α (eIF2α-P), and against Pgk1 as a loading control. For quantitative estimation of the level of eIF2α-P, for each sample the signal intensity of eIF2α-P was divided by that of Pgk1, and then normalised by the eIF2α-P/Pgk1 ratio of the unstarved WT cells. We found that deletion of *XRN1* led to reduced eIF2α-P levels under amino-acid starved conditions, as compared with that of the WT control strain (Figure 1B, lane 1 vs 3, Figure 1C), in agreement with the idea that Gcn2 activation was impaired. We observed that *XRN1* deletion also led to reduced eIF2α-P levels under non-starved conditions (Figure 1B, lane 6 vs 8, Figure 1C), suggesting that Xrn1 is also required for maintaining the basal level of Gcn2 activity.

**Figure 2.**
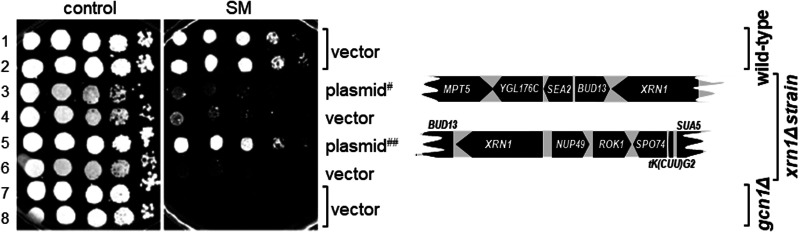
The SM^s^ phenotype of the *xrn1Δ* strain is complemented by a plasmid containing the intact *XRN1* gene. Wild-type strain BY4741, and isogenic *xrn1Δ* and *gcn1Δ* strains as indicated on the far right, were transformed with vector pRS425 or the tiling plasmids as indicated (YGPM19a16 (plasmid^#^), YGPM33c11 (plasmid ^##^)). Transformants were subjected to semi-quantitative growth assays as done in Figure1A, left panel. A map of the genes present in each tiling plasmid is shown.

**Figure 3.**
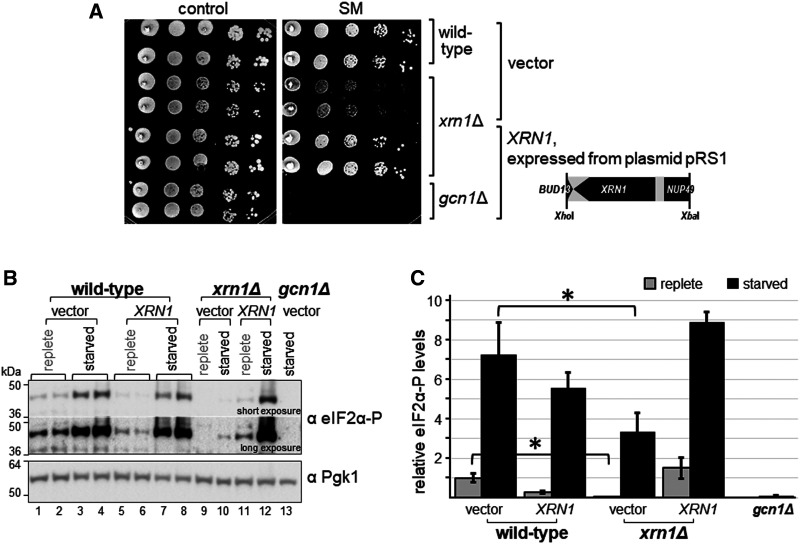
Plasmid borne *XRN1* reverts the SM^s^ of the *xrn1Δ* strain. (**A**) The indicated strains were transformed with vector alone or a plasmid containing *XRN1* under its endogenous promotor (plasmids pRS316 and pRS1). Then, independent transformants were subjected to a semi-quantitative growth assay as done in Figure 2. Plasmid pRS1 contains an *Xho*I-*Xba*I genomic DNA fragment that harbours the *XRN1* ORF in addition to fractions of the ORFs coding for *BUD13* and *NUP49,* as indicated in the figure. (**B**) Transformants from (**A**), as indicated, were subjected to immunoblotting as described in Figure 1B. Lanes 1 and 2, 3 and 4, 5 and 6, 7 and 8, respectively, are independent transformants. (**C**) The eIF2α-P levels were quantified as done in Figure 1C, using data from four biological replicates.

We next validated whether the SM^s^ was truly due to the intended deletion of *XRN1*, and not due to an ectopic mutation. For this, we first used two plasmids from the yeast genome tiling collection [40], a systematic library consisting of plasmids that each carry ∼10 kb fragments of the yeast genome. One plasmid contained the entire *XRN1* gene, while the other contained a truncated version of the gene (Figure 2A, schematic on the right side). In semi-quantitative growth assays, we found that a plasmid-borne genomic fragment containing full-length *XRN1* complemented the SM^s^ phenotype (Figure 2A, rows 1 and 2 vs 5), while a genomic fragment harbouring truncated *XRN1* did not (Figure 2A, rows 1 and 2 vs 3). To provide final evidence that the SM^s^ of the *xrn1Δ* strain is truly due to that missing gene, we subcloned a smaller genomic fragment that contained only the intact *XRN1* gene. A subsequent semi-quantitative growth assay revealed that plasmid borne *XRN1* was able to fully restore growth on starvation medium (Figure 3A). Next, we tested whether the impaired eIF2α-P levels of the *xrn1Δ* strain was complemented as well. As expected, we found that the plasmid containing the *XRN1* gene was able to restore the eIF2α-P levels, while empty plasmid (vector) did not (Figure 3B, lane 12 vs 3 and 4, Figure 3C). Taken together, our results suggest that Xrn1 is required for achieving WT eIF2α-P levels under starved as well as non-starved conditions.

### *In vivo* evidence that Xrn1 is in complex with Gcn1 and Gcn2

**Figure 4.**
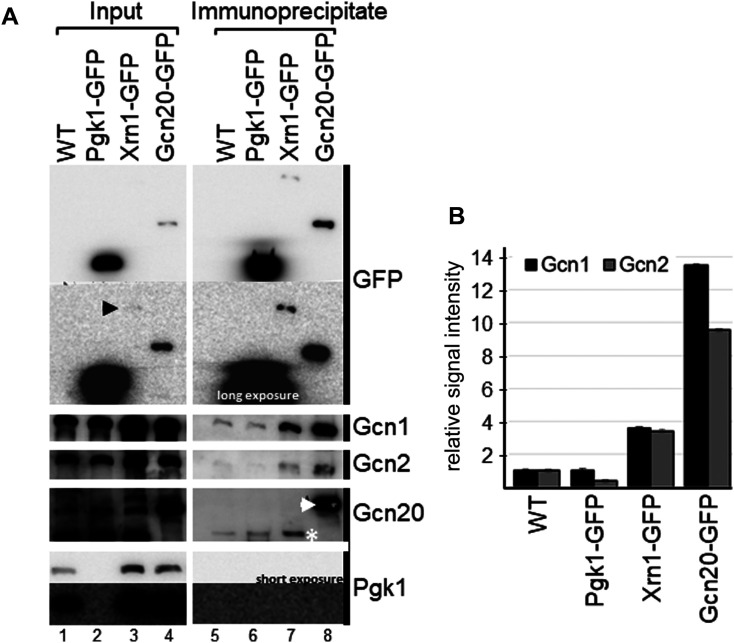
Xrn1 co-precipitates Gcn1 and Gcn2. (**A**) Cells expressing proteins with a C-terminal GFP tag as indicated, expressed from their endogenous chromosomal location and their endogenous promotor, were grown to exponential phase, and harvested. Whole cell extracts were generated and equal amounts of whole cell extract subjected to GFP-tag mediated co-immunoprecipitation assays. As input control, whole cell extract was loaded, representing 1% (lanes 1,2) or 3% (lanes 3,4) of the amount used in the co-precipitation experiments. Precipitates were subjected to SDS–PAGE and immunoblotting using antibodies against GFP, Gcn1, Gcn2, Gcn20, and Pgk1. Note that Pgk1-GFP migrates slower than Pgk1, and for that reason no signal can be detected for Pgk1-GFP in the membrane strip used for probing with the Pgk1 antibody. Pgk1-GFP can be readily detected with the GFP antibody. Black arrowhead points to the weak signal of Xrn1-GFP in the input lane. In the immunoprecipitate lanes (lanes 5–8) untagged Gcn20 and Gcn20-GFP are indicated with a white star and a white arrowhead, respectively. A representative of three independent experiments is shown. (**B**) Quantitation of Gcn1 and Gcn2 signals from (**A**) are shown. Gcn1 and Gcn2 signals from the precipitates were quantified relative to that of the input, and relative to the values of the WT precipitate.

Interactome studies found that Xrn1 and Gcn1 co-precipitate with the same bait proteins [33,35]. However, none of the interactome studies detected Gcn1 as prey when Xrn1 was used as bait, or *vice versa*. Therefore, we wanted to investigate whether Xrn1 and Gcn1 truly are members of the same protein complex. For this, a co-precipitation assay was performed using a strain expressing GFP-tagged Xrn1 from its own promotor and from its endogenous chromosomal location [41]. Cells were grown to exponential phase, cell extract generated, and then subjected to GFP-antibody mediated immunoprecipitation. The precipitates were resolved via SDS–PAGE, and then subjected to Western blotting using antibodies against the GFP tag, Gcn1, Gcn2, Gcn20, and Pgk1. We reproducibly found (three independent experiments) that the immuno-precipitates from the *XRN1-GFP* strain showed a stronger signal for Gcn1 and Gcn2 as compared with the untagged control strain or the *PGK1-GFP* control strain (Figure 4, lanes 7 vs 5 and 6), suggesting that Xrn1 is in complex not only with Gcn1, but also with Gcn2. Gcn20 was not reproducibly found in the Xrn1-GFP precipitate, suggesting that if Gcn20 is part of the complex it is only weakly bound. Pgk1 is a highly abundant housekeeping gene not known to bind to Gcn1 or Gcn2. Even after a long exposure, Pgk1 was not detectable in the immuno-precipitates from the WT strain (Figure 4, lane 5), nor was Pgk1 detectable in the Xrn1-GFP or Gcn20-GFP precipitates (Figure 4, lanes 7 and 8), suggesting that, at least for Pgk1, un-specifically bound proteins were efficiently removed. Thus, our findings support the idea that Xrn1 specifically co-precipitates Gcn1 and Gcn2, suggesting that Xrn1, Gcn1 and Gcn2 reside in the same protein complex.

### Evidence that *XRN1* deletion affects the GAAC response upstream of Gcn4 translational regulation

**Figure 5.**
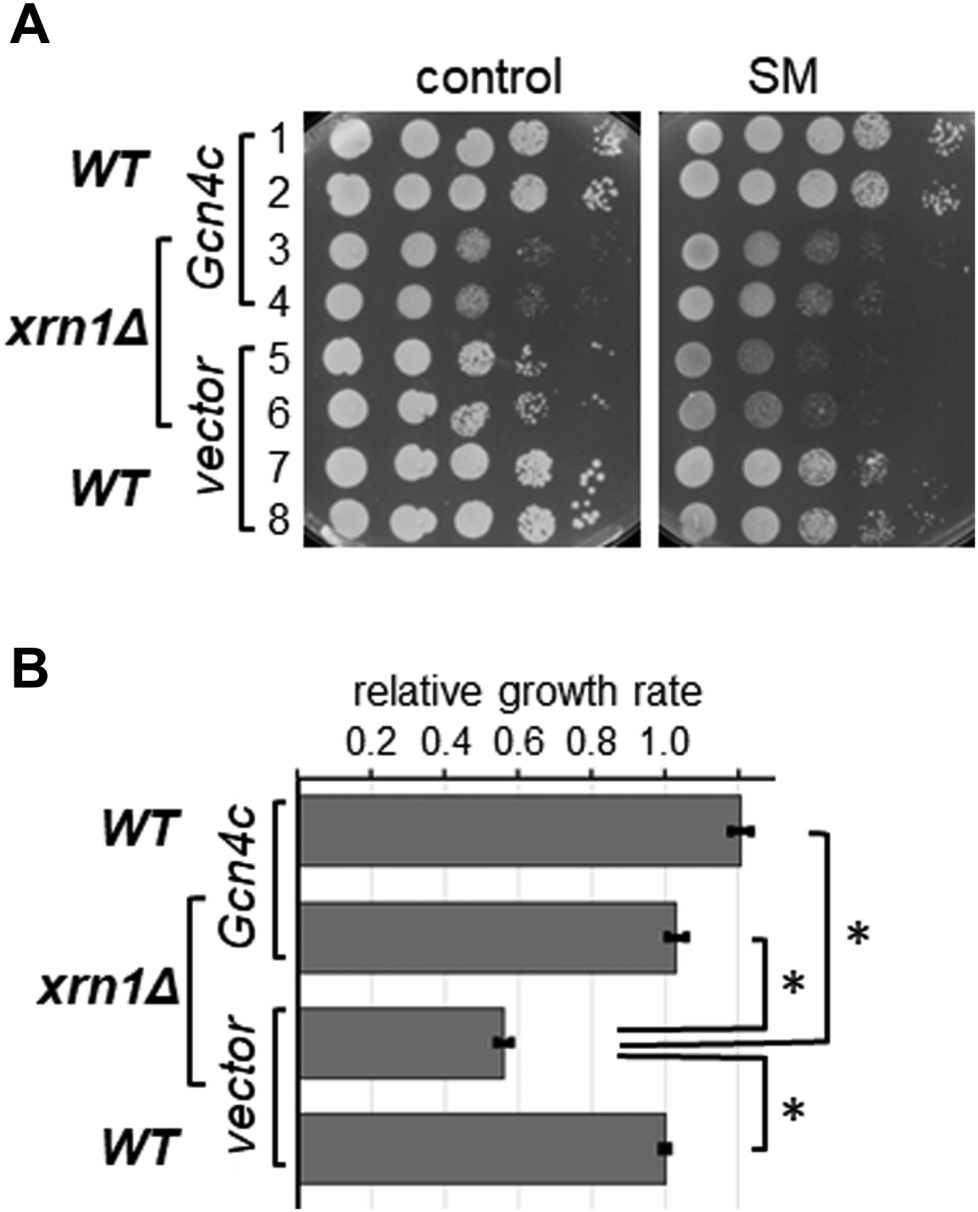
Constitutively expressed Gcn4 reverts the SM^s^ phenotype elicited by the *XRN1* deletion. (**A**) The wild-type strain and its isogenic *xrn1Δ* strain were transformed with vector alone (YCp50) or a plasmid harbouring *GCN4* under its own promoter but lacking the inhibitory uORFs in its 5′ mRNA untranslated region (dubbed *GCN4^c^*) (p238). Transformants were then subjected to semi-quantitative growth assays as done in Figure 1A. (**B**) Quantitative evaluation of the strains’ sensitivity to SM in (**A**). As outlined in more detail in the materials and methods section, the growth defect of the strains seen under non-starvation conditions (control) was accounted for when determining the growth rates on the starvation medium (SM). The growth rates were then plotted on a bar graph, relative to that of the wild-type strain harbouring vector alone. Error bars depict the standard error, and stars indicate significant differences between values (Student's *t*-test, *P* ≤ 0.05).

If the SM^s^ phenotype of the *xrn1Δ* strain is truly due to reduced eIF2α-P levels, and concomitant reduced translational depression of Gcn4, then constitutively increased *GCN4* translation should revert the SM^s^ phenotype. To test this, we introduced into the *xrn1Δ* strain, and into the isogenic WT strain as control, a plasmid harbouring *GCN4* under its own promoter but lacking the inhibitory uORFs in its 5′ mRNA untranslated region (dubbed *GCN4^c^*). This well-characterised plasmid leads to the constitutive high abundance of Gcn4 in the cell [42]. In subsequent semi-quantitative growth assays, under non-starved conditions, deletion of *XRN1* led to a growth defect (Figure 5A, control plate, rows 5,6 vs 7,8) as reported previously [43]. While Gcn4^c^ did not affect the growth rate of the WT strain (Figure 5A, control plate, rows 7,8 vs 1,2), *GCN4^c^* exacerbated the growth defect of the *xrn1Δ* strain (Figure 5A, control plate, rows 3,4 vs 5,6). In contrast with that, under starved conditions, Gcn4^c^ improved the growth of the *xrn1Δ* strain (Figure 5A, SM plates, rows 3,4 vs 5,6). Next, we quantitatively evaluated the growth rates of each strain on starvation medium, relative to that on the control plates. This allowed us to take into account the growth differences of strains on the control plate (non-starved conditions), i.e. to take into account growth differences not caused by SM. This permitted a more objective evaluation on the severity of the SM^s^ phenotype (Figure 5B). The data suggested that, on starvation medium, Gcn4^c^ enhanced the growth rate of the WT strain slightly, though this difference was not statistically significant (Figure 5A, rows 1 and 2 vs 7 and 8; Figure 5B, compare the bottom and top bars). In contrast with that, Gcn4^c^ almost doubled the growth rate of the *xrn1Δ* strain on starvation medium (Figure 5A, rows 5 and 6 vs 3 and 4; Figure 5B, compare the two middle bars). In fact, when normalising for the growth defect on the control plates, the growth rate of the *xrn1Δ* strain harbouring Gcn4^c^ was not statistically different from that of the WT strain containing vector alone or Gcn4^c^ (Figure 5B, compare the top two bars and the bottom bar). It shall be noted that these conclusions were made under the assumption that Gcn4^c^ led to the same high Gcn4 protein levels in WT and *xrn1Δ* strains. Nevertheless, the findings suggested that the SM^s^ phenotype of the *xrn1Δ* strain can be suppressed by overexpression of Gcn4, in agreement with the idea that removal of *XRN1* leads to a defect upstream of Gcn4 translational regulation.

### *XRN1* deletion does not revert the slg^−^ phenotype elicited by constitutively active Gcn2

Reduced eIF2α-P levels could be the result of impaired Gcn2 activation, or the result of increased activity of protein phosphatase 1 (PP1, encoded by *GLC7*) de-phosphorylating eIF2α-P [44]. To test whether the PP1 activity was enhanced in *xrn1Δ* strains, we took advantage of mutations that render Gcn2 constitutively active.

The Gcn2 E803V substitution renders Gcn2 constitutively active [37], but this Gcn2 variant (dubbed Gcn2^c^) still requires Gcn1 to become constitutively active [45,46]. Activated Gcn2^c^ leads to eIF2α hyper-phosphorylation, thereby dramatically impacting on global protein synthesis, and consequently leading to a growth defect even under non-starved conditions. Thus, this slow growth (slg^−^) phenotype is indicative of Gcn2 hyper-activity. Since Gcn2^c^ only requires to be activated once for its consequent permanent activation, we reasoned that if Xrn1 impairs — but not fully blocks — Gcn1-mediated Gcn2 activation, then the activity of Gcn2^c^ should hardly be affected in an *xrn1Δ* strain. However, if *XRN1* deletion leads to enhanced PP1 activity, this should counteract Gcn2^c^ mediated eIF2α hyper-phosphorylation, visible by the reversion of the slg^−^ phenotype.

**Figure 6.**
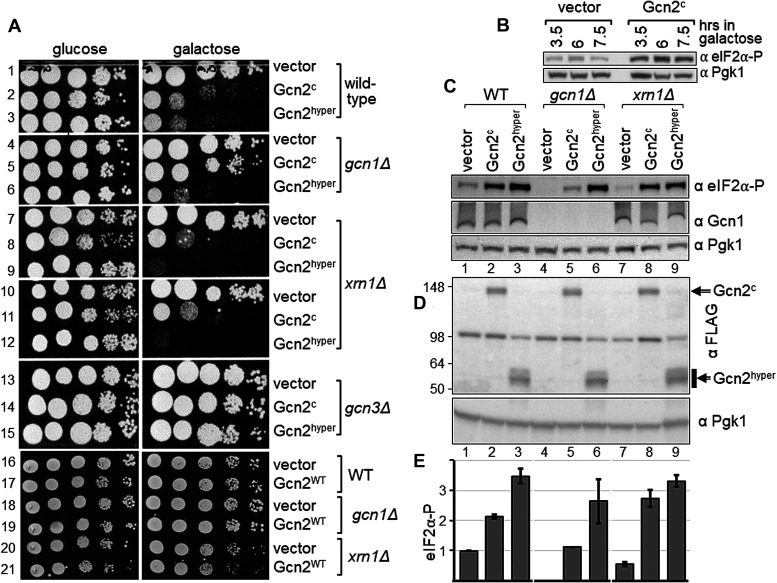
Deletion of *XRN1* does not revert the growth defect associated with constitutively active Gcn2. (**A**) Strains deleted for the indicated gene, or isogenic wild-type strain BY4741, were transformed with vector alone (pEMBLyex4), or a plasmid expressing from a galactose inducible promotor constitutively active Gcn2^c^ or Gcn2^hyper^ (pDH114, pHQ1213), or wild-type Gcn2 (Gcn2^WT^, pDH103). Transformants were then subjected to semi-quantitative growth assays as done in Figure 2, but on medium containing glucose or galactose. (**B**) The level of phosphorylated eIF2α was determined via western blotting as described in Figure 1B, except that *xrn1Δ* cells were grown to exponential phase in medium containing raffinose, and then galactose added. Cells were harvested 3.5, 6, and 7.5 h thereafter. (**C**) The level of phosphorylated eIF2α, as well as the level of endogenous Gcn1 was determined as described in (**B**), using antibodies against eIF2α, Gcn1, and Pgk1, with exposure to galactose for 6 h before harvesting. For more detail see text. (**D**) The level of FLAG-tagged Gcn2^c^ and Gcn2^hyper^ was determined as described in (**C**), using antibodies against FLAG, and Pgk1. (**E**) The eIF2α-P signals in (**C**) were quantified relative to that of wild-type containing vector alone, as done in Figure1C. The average of at least four biological replicates is shown, as well as the standard error.

To test this, we conducted semi-quantitative growth assays using the WT strain BY4741 and isogenic strains deleted for *GCN1*, *GCN3* and *XRN1*, respectively, that each contained vector alone or a plasmid expressing Gcn2^c^ from a galactose inducible promotor. As expected, the growth defect elicited by Gcn2^c^ was apparent in the WT strain but not in the *gcn1Δ* strain (Figure 6A, row 2 vs 1, row 5 vs 4). In a *gcn3Δ* strain eIF2α-P is unable to inhibit its GEF exchange factor eIF2B [2], meaning that eIF2α-P is unable to hamper protein synthesis despite of its hyper-phosphorylation. Accordingly, as expected, Gcn2^c^ was unable to cause a slg^−^ phenotype in the *gcn3Δ* strain (Figure 6A, row 14 vs 13). In the *xrn1Δ* strain, we found that Gcn2^c^ still elicited a growth defect that was comparable to that of the WT strain (Figure 6A, row 8 vs 7, 11 vs 10, 2 vs 1). This is in agreement with the idea that *XRN1* deletion does not lead to enhanced PP1 activity.

A fragment encompassing the Gcn2 protein kinase domain (amino acids 591–1010), and harbouring the R794G;F842L double substitution, is constitutively active [47]. The mutations bypass the requirement of Gcn1 for this protein kinase domain to become constitutively active [47], and therefore this constitutive Gcn2 fragment is dubbed Gcn2^hyper^. As expected, in a *gcn1Δ* strain, Gcn2^hyper^ elicits a slg^−^ phenotype in contrast with Gcn2^c^ (Figure 6A, rows 6 vs 5 vs 4), while in a *gcn3Δ* strain Gcn2^hyper^ did not elicit a slg^−^ phenotype (Figure 6A, rows 15 vs 14 vs 13). We found that Gcn2^hyper^ caused a growth defect in the *xrn1Δ* strain, as found for the WT and *gcn1Δ* strain (Figure 6A, rows 9 vs 7, 12 vs 10, 3 vs 1, 6 vs 4).

As a control, we repeated the experiment but used the Gcn2 WT version. Gcn2^WT^ needs a signalling cue such as amino acid starvation to become activated. As expected, on medium containing galactose no growth defect can be observed (Figure 6A, rows 16–21), given that under these conditions Gcn2 is overexpressed but has not been activated.

To test whether the observed impaired growth was truly due to hyperactive Gcn2, we scored for the levels of eIF2α phosphorylation. Experimental procedures require exponentially growing cells for scoring eIF2α phosphorylation levels. However, constitutively active Gcn2 elicits a growth defect, and Gcn2^hyper^ barely allows any growth. For that reason, we grew cells first to exponential phase (to an OD of 0.4, for ∼15 h) in medium containing 2% raffinose (w/v) as carbon source, before adding galactose (2% w/v final) to induce expression of Gcn2^c^ and Gcn2^hyper^. Raffinose was used as — in contrast with glucose — it does not prevent galactose-mediated promotor induction. We found that growth for 3.5 h in galactose medium already led to eIF2α-P levels in strains expressing Gcn2^c^ (Figure 6B). Therefore, for our experiments we chose to expose cells for 6 hrs to galactose before harvesting. As expected, we found that in the WT strain Gcn2^c^ led to increased eIF2α-P levels, and Gcn2^hyper^ led to even higher levels (Figure 6C, lane 1 vs 2 vs 3, Figure 6E). Also, as expected, in the *gcn1Δ* strain only Gcn2^hyper^ elicited high eIF2α-P levels (Figure 6C, lane 6 vs 1, Figure 6E), while Gcn2^c^ lead to eIF2α-P levels that were similar to the basal eIF2α-P levels in the WT strain (Figure 6C, lane 5 vs 1, Figure 6E). In *xrn1Δ* strains Gcn2^c^ and Gcn2^hyper^ elicited increased eIF2α-P levels comparable to those in the WT (Figure 6C, lane 8 vs 2, lane 9 vs 3; Figure 6E). This suggests that the observed impaired growth was truly due to enhanced eIF2α-P phosphorylation.

Taken together, these findings are in agreement with the idea that in an *xrn1Δ* strain the reduced eIF2α-P levels are not due to enhanced PP1 activity, and that Xrn1 is required for full or efficient Gcn2 activation, directly or indirectly.

### Xrn1-ribosome interaction is not required for growth on starvation medium

**Figure 7.**
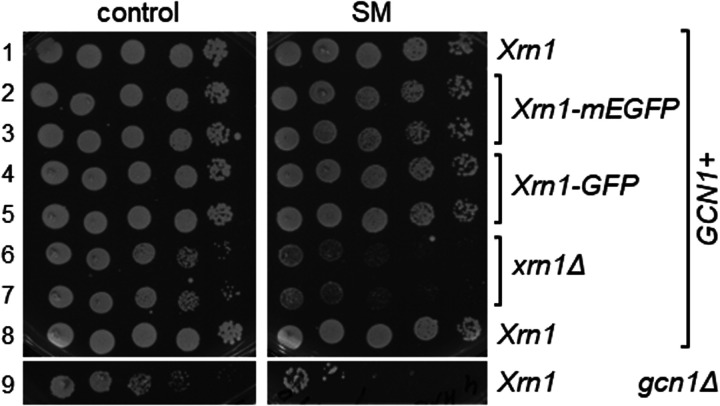
Xrn1 unable to bind to ribosomes is still able to complement the SM^s^ phenotype of a *xrn1Δ* strain. Strains harbouring Xrn1 (strain BY4742 in row 1, and BY4741 in row 2), Xrn1 containing an internal mEGFP tag that sterically hinders ribosome binding (Xrn1-mEGFP), or C-terminally GFP-tagged Xrn1, and a *xrn1Δ* and *gcn1Δ* strain, were subjected to semi-quantitative growth assays as done in Figure 1A. Strains with mating type a are Met auxotropic, and with mating type α are Lys auxotrophic. The wild-types differing in the mating type and the according auxotrophies (rows 1 and 8) did not show differences in growth on SM, implying that the difference in mating type and auxotrophies did not affect the sensitivity to SM.

Xrn1 binds to ribosomes [38], raising the question whether this interaction is required for promoting full Gcn2 activation. To test this, we used a strain that expresses from its native chromosomal location an Xrn1 protein incapable of ribosome-binding [38]. This was achieved by an in-frame insertion of the monomeric enhanced green fluorescent protein (mEGFP) into XRN1 (after Ser-235), which sterically hinders the interaction of Xrn1 with the ribosome [38]. Strains containing Xrn1-mEGFP did not show a growth defect as found for a *xrn1* deletion strain (Figure 7, left panel, rows 2 and 3 vs 6 and 7), but instead grew as well as the WT strain (Figure 7, left panel, rows 2 and 3 vs 1 and 8), suggesting that the mEFGP insertion did not affect Xrn1 function, at least not to a large extent [38], and that Xrn1-mEGFP was sufficiently expressed. We found that on SM media, the strain harbouring Xrn1-mEGFP grew as well as the strain containing endogenous WT Xrn1, or C-terminally GFP-tagged Xrn1 (Figure 7, right panel, rows 2 and 3 vs 1 and 8 vs 4 and 5). This suggested that Xrn1-ribosome interaction is not necessary for mediating efficient Gcn2 activation.

### The Xrn1 3′ → 5′ exonuclease activity is required for growth on starvation medium

**Figure 8.**
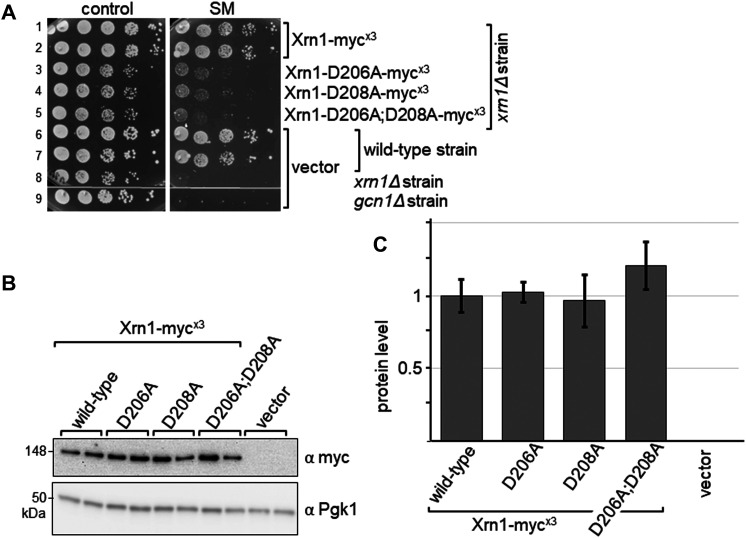
Enzymatically inactive Xrn1 is unable to complement the SM^s^ phenotype of a *xrn1Δ* strain. (**A**) Strains expressing proteins as indicated were subjected to a semi-quantitative growth assays as done in Figure 2. (**B**) Transformants from (**A**), as indicated, were subjected to immunoblotting as described in Figure 1B, except that the cells were not starved, using antibodies against the myc tag present at the C-terminus of Xrn1, and against Pgk1. Lanes 1 and 2, 3 and 4, 5 and 6, 7 and 8, 9 and 10, respectively, are independent transformants. (**C**) The Xrn1 protein level was quantified relative to that of wild-type Xrn1, as done in Figure1C. Quantifications were performed from four biological replicates.

To test whether the Xrn1 enzymatic activity is required for conferring growth on starvation medium, we generated plasmid-borne *XRN1* expressed from its native promotor and harbouring a triple-myc tag at its C-terminus, and Xrn1 carrying amino acid substitutions known to be essential for 3′ → 5′ exonuclease activity [48]. These D206A and D208A substitutions, singly or in combination, have been shown previously to abolish enzymatic activity [48]. The triple-myc tagged Xrn1 was able to fully suppress the growth defect of an *xrn1Δ* strain, as well as fully restore growth on starvation medium, suggesting that the triple-myc tag did not affect Xrn1 function (Figure 8A, rows 1,2 vs 6,7 vs 8). The mutated Xrn1 proteins were expressed at least as well as WT Xrn1 (Figure 8B,C). Yet, on starvation plates *xrn1Δ* strains containing mutated Xrn1 clearly displayed a SM^s^ phenotype (Figure 8A, rows 3–4 vs 1 and 2). This suggests that the Xrn1 3′ → 5′ exonuclease activity is required for conferring full growth on starvation medium.

## Discussion

The GAAC pathway is best known for its relevance in coping with and overcoming amino acid starvation. In this pathway, Gcn2 senses amino acid availability [1,2]. For this, Gcn2 must directly bind to its effector protein Gcn1. Gcn1 belongs to the family of HEAT repeat proteins. Since some of these HEAT repeat proteins have been reported to be scaffold proteins [16,19], this raises the intriguing possibility that Gcn1 is a hub for other proteins to bind and modulate Gcn2 activity according to the cell's needs. In fact, in interactome studies many proteins have been found that are potentially in complex with Gcn1 [32–35]. In these studies, Xrn1 was reported to co-precipitate along with Gcn1 with the same bait proteins [33,35]. We here have shown that GFP-tagged Xrn1 co-precipitated Gcn1 *in vivo* as well as Gcn2, raising the possibility that all three proteins, Gcn1, Gcn2 and Xrn1, can reside in the same complex. Supporting this idea, Gcn1 and Gcn2 directly interact with each other [6]. In contrast with Gcn1, the large-scale interactome studies did not detect Gcn2 in Xrn1 containing complexes, possibly because Gcn2 is hard to detect due to its low abundance, or because the relevant protein–protein interactions were too weak to sustain the experimental procedures used in these interactome studies.

Gcn1 and Gcn2 directly contact each other [6], and each can associate with ribosomes [1,2] as found for Xrn1 [38], raising the possibility that Xrn1–Gcn1 and/or Xrn1–Gcn2 interaction was bridged by the ribosome. Though, given the size of the ribosome, it seems unlikely that standard immunoprecipitation protocols could precipitate ribosomes. Nevertheless, our studies do suggest that Gcn1, Gcn2, and Xrn1 reside in the same complex.

In this study, we have obtained several lines of evidence that Xrn1 is required for full Gcn2 activation. A *xrn1Δ* strain showed reduced ability to grow on starvation medium. This correlated with reduced levels of phosphorylated eIF2α (eIF2α-P), in agreement with the idea that Gcn2 activation was impaired. In an alternative scenario, the removal of Xrn1 may have stimulated the phosphatase PP1, leading to enhanced rates of eIF2α-P dephosphorylation. Though, thus far no link between Xrn1 and phosphatases has been reported. Also, we here have found that constitutively active Gcn2 elicited a growth defect that was not reverted by the removal of Xrn1, nor was the eIF2α hyper-phosphorylation dampened, which would argue against a scenario involving enhanced eIF2α-P dephosphorylation.

Increased eIF2α-P levels are required to initiate the next step in the GAAC signalling pathway, which is the enhanced translation of the *GCN4* mRNA. In agreement with the idea that *XRN1* deletion impairs the GAAC at the level of eIF2α/eIF2α-P, we found that constitutively translated *GCN4* (Gcn4^c^) rescued the SM^s^ phenotype of a *xrn1Δ* strain.

Gcn4^c^ did not rescue the slg^−^ phenotype associated with the deletion of *XRN1*, but instead seemed to have exacerbated this growth defect. While Gcn4 is a transcriptional regulator determining the rate of transcription of specific genes [2], Xrn1 is involved in mRNA decay and quality control, as well as translational regulation through modifying the abundance of specific mRNA species via miRNA, siRNA, and lncRNA [36]. Hence, the exacerbation effect may have been due to certain mRNAs being targeted by both Gcn4 and Xrn1. Further studies would be necessary to investigate which mRNAs are affected by both Gcn4 and Xrn1, and may help reveal new links/cross-talks between the GAAC pathway and Xrn1 mediated processes.

While Gcn1-ribosome and Gcn2-ribosome interaction are each necessary for Gcn2 activation [6–8], our findings seem to indicate that Xrn1-ribosome interaction is not required for promoting Gcn2 activation. It will be interesting to determine whether direct Xrn1–Gcn1 or Xrn1–Gcn2 interaction is necessary for promoting Gcn2 activation. Since Xrn1 plays a role in resolving stalled ribosomes [21], and since a link has been reported between Gcn2 and ribotoxic stress [14], it will be interesting to investigate whether the Xrn1/Gcn1 axis is relevant for resolving stalled ribosomes and/or the ribotoxic stress pathway.

Xrn1's function in mRNA decay and quality control requires its exonuclease activity. Our findings suggest that the Xrn1 exonuclease activity is also required to promote full Gcn2 activation. Given that the recognition of the starvation signal and the concomitant increase in eIF2α phosphorylation involves proteins already present in the cell (Gcn1, Gcn2, eIF2), how can Gcn2 activation be promoted by Xrn1's function in mRNA decay and quality control?

In one scenario, efficient Gcn1-mediated Gcn2 activation could require the Xrn1 protein to be in close proximity to Gcn1 and Gcn2. Supporting this idea, Xrn1 is physically in the same protein complex as Gcn1 and Gcn2. Xrn1 may be required for promoting the proper orientation of Gcn1 and Gcn2 on the ribosome, in order to ensure that Gcn2 has access to the starvation signal and/or to its substrate eIF2α. While Xrn1-ribosome interaction is not required for promoting full Gcn2 activation, it is still possible that Xrn1 exerts its role via Xrn1–Gcn1 and/or Gcn1–Gcn2 interaction.

**Figure 9.**
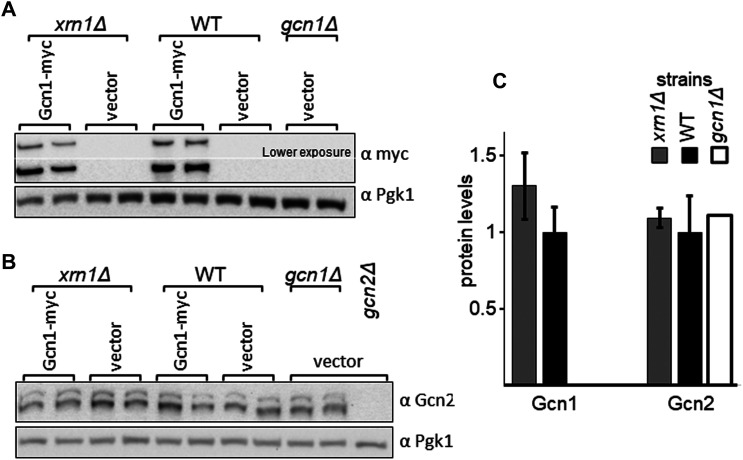
Deletion of *XRN1* does not lead to reduced levels of Gcn1 or Gcn2. (**A,B**) Strains harbouring empty vector or a plasmid expressing myc tagged Gcn1 from its native promotor, as indicated, were grown in liquid medium to exponential phase. Cells were harvested, and the whole cell extract used for immunoblotting as done in Figure 8B, using antibodies against the myc tag, Gcn2, and Pgk1 as loading control. (**C**) The Gcn1 and Gcn2 protein levels were quantified relative to that of the wild-type, as done in Figure1C. Quantifications were performed from four biological replicates.

In a second scenario, *XRN1* deletion may have led to reduced levels of proteins relevant for eIF2α phosphorylation, such as the proteins Gcn1 and Gcn2. This may be due to enhanced protein degradation, reduced translation *per se*, or due to decreased *GCN1* or *GCN2* mRNA levels. However, *XRN1* deletion has not been reported yet to promote protein degradation. *XRN1* deletion has been reported to affect the levels of specific mRNAs [49], however, Xrn1 is involved in mRNA decay [50], as well as miRNA, siRNA and lncRNA-mediated gene repression [36] aimed to dampen the translation of specific mRNAs. This would mean that *XRN1* deletion would lead to increased — rather than decreased — mRNA levels or mRNA translation. Supporting this notion, past studies suggest that Gcn2 and Gcn1 mRNA levels are not increased in *xrn1Δ* strains [49]. Also, here we have not found any indication for reduced Gcn1 or Gcn2 levels in *xrn1Δ* strains (Figure 9).

A third scenario is based on the fact that Xrn1 is involved in tRNA quality control [36]. Aberrant tRNAs may accumulate in an *xrn1Δ* strain, though one would expect that these would enhance Gcn2 activation as long as they can be detected by Gcn2.

A fourth scenario is based on the fact that Xrn1 is relevant for the processing and maturation of rRNA and thus ribosome biogenesis [36]. In *xrn1Δ* strains Gcn1 and Gcn2 may be unable to properly contact the resulting ‘faulty’ ribosomes. This could hamper the efficient detection of the starvation signal, and dampen Gcn2 activation.

The fifth scenario is based on the fact that Xrn1 has been reported to have an additional biological role that is unrelated to its role in RNA metabolism, which is its function in meiosis [48]. This raises the possibility that Xrn1 may have more not-yet-discovered non-canonical functions, and one of these could be the modulation of Gcn2 activation.

Finally, we cannot exclude the possibility that Xrn1's actions in the cell very indirectly affect the level of Gcn2 activation. Nevertheless, no matter how indirect, Xrn1 deletion hampering Gcn2 activation could be a physiologically relevant mechanism for finetuning Gcn2 activity to the cell's needs.

In this work, we have provided evidence that Xrn1 is required for the full activity of Gcn2, directly or indirectly. This suggests a potential new link between RNA metabolism and the GAAC signalling pathway. While it is not known yet whether its presence in the Gcn1/Gcn2 complex is relevant for promoting GAAC activity, our studies do suggest that Xrn1-ribosome interaction is not required for mediating full Gcn2 activation. It is tempting to speculate that through regulation of the Xrn1 exonuclease activity, and/or through Xrn1 shuttling in or out of the Gcn1/Gcn2 complex, the cell controls the threshold level for GAAC stimulation and/or the intensity of the GAAC response. Xrn1-mediated adjustment of the GAAC may occur in response to environmental or internal stimuli, such as the level of aberrant RNAs. In fact, studies suggest that Xrn1 activity can be regulated in response to cues, e.g. via sequestration to the eisosome under conditions of glucose deprivation, or through the accumulation of aberrant metabolic intermediates [36,51]. These intriguing possibilities warrant subsequent in-depth studies to unravel the mechanism by which Xrn1 promotes full eIF2α-P levels in the cell, and whether Xrn1 association with the Gcn1/Gcn2 complex is required for regulating the GAAC.

## Materials and methods

### Yeast strains and plasmids

Yeast strains and plasmids used in this study are listed in Tables 1 and 2. Empty vectors used were pEMBLyex4 [53], pRS316 [54], pRS425 [55], and YCp50 [56].

**Table 1.** Strains used in this study

Strain	Genotype	Source
Genetic background H1511		
H1511	*MATα ura3-52 trp1-63 leu2-3,112, GAL2^+^*	[52]
H2556	Same as H1511 but *gcn1Δ*	[6]
H2557	Same as H1511 but *gcn2Δ*	[6]
Genetic background BY4741 or BY4742		
BY4741	*MATa his3Δ1 leu2Δ0 met15Δ0 ura3Δ0*	Dharmacon
BY4742	*MATα his3Δ1 leu2Δ0 lys2Δ0 ura3Δ0*	Dharmacon
*xrn1Δ* strain	Same as BY4741 but *xrn1Δ*::KanMX4	Dharmacon
Xrn1-mEGFP strain	Same as BY4742 but mEGFP is inserted into the *XRN1 ORF* after the Ser-S235 triplet codon	[38]
EMSY6053-3-1	Same as BY4741 but *gcn2Δ::HisG*	[23]
PGK1-GFP strain	Same as BY4741 but *PGK1-GFP*^a^	Thermo Fisher
GCN20-GFP strain	Same as BY4741 but *GCN20-GFP*^a^	Thermo Fisher
XRN1-GFP strain	Same as BY4741 but *XRN1-GFP*^a^	Thermo Fisher

a Epitope tag at the C-terminus of the ORF.

**Table 2.** Plasmids used in this study

Plasmid	Gene	Selectable marker	Vector	Source
Yeast gene fusions, under Galactose inducible promotor				
pDH114	*Flag-His_6_*^a^*-GCN2-E803V* (coding for Gcn2^b^)	*Amp^R^, URA3, leu2d*	pEMBLyex, 2µ	[45]
pHQ1213	*Flag-His_6_*^a^*-GCN2[591-1010]*^c^*-R794G,F842L* (coding for Gcn2^hyper^)	*Amp^R^, URA3, leu2d*	pEMBLyex, 2µ	[47]
pDH103	*Flag-His_6_* ^a^ *-GCN2*	*Amp^R^, URA3, leu2d*	pEMBLyex, 2µ	[47]
Yeast genes, under own promotor				
pRS1	*XRN1*	*Amp^R^, URA3*	pRS316, CEN/ARSH4	This study
pRA1001	*XRN1*	*Amp^R^, URA3*	pRS316, CEN/ARSH4	This study
pRA1002	*XRN1-myc^x3^*	*Amp^R^, URA3*	pRS316, CEN/ARSH4	This study
pRA1003	*xrn1-D206A-myc^x3^*	*Amp^R^, URA3*	pRS316, CEN/ARSH4	This study
pRA1004	*xrn1-D208A-myc^x3^*	*Amp^R^, URA3*	pRS316, CEN/ARSH4	This study
pRA1005	*xrn1-D206A;D208A-myc^x3^*	*Amp^R^, URA3*	pRS316, CEN/ARSH4	This study
p238	*GCN4*	*Amp^R^, URA3*	YCp50, ARS1/CEN4	[42]
Tiling collection plasmids, with yeast genome fragments				
pGP564	*empty vector*	*Amp^R^, LEU2*	pGP564, 2µ	Dharmacon
YGPM33c11	Genome fragment contains: *BUD1,*^d^ *XRN1, NUP49, ROK1, SPO74, tK(CUU)G2, SUA5*^d^			Dharmacon
YGPM19a16	Genome fragment contains: *MPT5,*^d^ *YGL177W,*^e^ *YGL176C, SAE2, BUD13, KEM*^d^			Dharmacon

a Epitope tag at the N-terminus of the ORF.

b The *GCN4* 3’ UTR lacks the uORF, leading to constitutive *GCN4* translation.

c Numbers in brackets indicate amino acids encoded by the respective gene.

d ORF truncated.

e ORF intact, but up/downstream regulatory elements may be missing.

Plasmid pRS1 harbouring *Xrn1* under its own promotor was constructed by digesting plasmid YGPM33c11 (Dharmacon) with *Xho*I and *Xba*I, and inserting the resulting 6.6 kb long DNA fragment into the similarly digested plasmid pRS316.

In plasmid pRS1, the *Not*I site in the multiple cloning site was removed (GCGGCCGC was replaced by GCGGCCaC) commercially (Genscript, U.S.A.), yielding pRA1001. Then — just upstream of the *XRN1* stop codon — the sequence GCG GCC GCA TTG ggt ggt gga GAA GAA CAA AAG TTG ATT TCT GAA GAA GAC TTG ggt ggt gga ggt ggt GAA CAA AAG TTG ATT TCT GAA GAA GAC TTG ggt ggt gga ggt ggt GAA CAA AAG TTG ATT TCT GAA GAA GAC TTG TTG AGA AAG AGA GCG GCC GCT was added commercially, which codes for a 3× myc tag flanked by *Not*I sites (Genscript, U.S.A.), yielding pRA1002. In pRA1002 the D206 and D208 substitutions, singly and in combination, were introduced commercially via site-directed mutagenesis (Genscript, U.S.A.) resulting into pRA1003, pRA1004, pRA1005, respectively.

### Yeast culture conditions

Cultures were grown in YPD media or in synthetic dextrose media containing the appropriate supplements to cover auxotrophies. To induce expression of genes driven by the galactose inducible promoter, 2% (w/v) galactose was used as carbon source instead of 2% (w/v) glucose. When grown in liquid media, cultures were shaken at 160 rpm. Solid medium contained 2% agar. All *S. cerevisiae* cultures were grown at 30°C unless stated otherwise.

For semi-quantitative growth assays, yeast liquid overnight cultures were subjected to four 10-fold serial dilutions using synthetic dextrose medium lacking supplements and a carbon course. Then, 5 μl of the overnight cultures and of the dilutions were transferred to solid medium. The plates were incubated at 30°C, and the growth documented using a conventional document scanner. When strains showed growth differences on control plates — making it more difficult to determine the effect of SM on cell growth — the growth on SM plates was evaluated quantitatively as published previously [57]. Briefly, for each strain on a plate, for each of the five dilutions a growth score was given from 0 to 10, with score 10 being full growth. Then, for each plate and strain, the sum of the five growth scores was determined, resulting in the overall growth score. For each strain, the overall growth score on the starvation plate was divided by that of the same strains growing on the control plate. The resulting adjusted growth score was divided by that of the WT strain expressing GST alone, leading to the relative growth rate. Relative growth rates were then plotted in a bar graph along with the standard error.

### Generating cell pellets from exponentially growing yeast cells

For western blotting assays, cells were grown and harvested as published previously [58]. Briefly, a 250 ml flask containing 50 ml medium was inoculated with a fresh yeast overnight culture and incubated at 160 rpm and 30°C. At OD_600 nm_ between 0.9 and 1, the cells were subjected to formaldehyde treatment for 1 h (final concentration 1%), and then centrifuged at 2000***g*** for 3 min. Cell pellets were immediately stored at −80°C.

For co-precipitation assays, a 1 l indented flask containing 300 ml of liquid medium was inoculated with a fresh yeast overnight culture and incubated at 160 rpm and 30°C. At OD_600 nm _= 1–1.5, the cells were pelleted by centrifugation at 2000***g*** for 5 min at 4°C, the pellet re-suspended with 5 ml of ice-cold breaking buffer (BB, 30 mM HEPES-KOH, pH 7.4, 50 mM KCl, 10% glycerol) containing protease inhibitors (1 mM PMSF, 10 µg/ml Pepstatin, 1 µg/ml Aprotinin, 1 µg/ml Leupeptin and 5 mM β-mercaptoethanol), transferred to a 13 ml round bottom tube, and then re-pelleted by centrifugation at 2000***g*** for 5 min at 4°C. The pellets were immediately frozen at −80°C.

### Generating whole cell extracts

For western blotting, cells were lysed using sodium hydroxide, as published previously [58]. Briefly, cell pellets were resuspended in sodium hydroxide solution, and the cells pelleted again to remove the solution. The pellet was then resuspended in 2× protein loading buffer (0.1% (w/v) bromophenol blue, 4% (w/v) SDS, 100 mM Tris–Cl (pH 6.8), 20% (v/v) glycerol and 1.47 M β-mercaptoethanol), and subjected to heat treatment at 80°C to fully dissolve the pellet.

For co-precipitation assays, one pellet volume of ice-cold BB containing protease inhibitors (see above) and one pellet volume of acid washed glass beads were added to the cell pellet. The samples were subjected to vortexing 10 times at high speed for 30 s, alternating with 30 s intervals in an ice-water mix, as described earlier [58,59]. The cell debris was removed by centrifugation at 2000***g*** for 5 min at 4°C, the supernatant transferred to a 1.5 ml tube, followed by a spin at 19 000***g*** for 10 min at 4°C. The supernatant was collected in fresh tubes and the protein concentration determined using the Bradford protein estimation method [60].

### Co-immunoprecipitation assays

Whole cell extracts (1 mg) were incubated with 20 µl (100% bed volume) of protein A resin (Sigma–Aldrich), in a total volume of 480 µl, for 1 h at 4°C. The samples were then centrifuged at 100***g*** for 1 min at 4°C, and 440 µl of the supernatant was transferred to a fresh tube. Then, 400 µl of the supernatant was transferred to a tube containing 20 µl bed volume of anti-GFP antibodies covalently linked to sepharose beads (Abcam, #ab69314, coated with 5% BSA prior to usage), and incubated for 2 hrs at 4°C. After centrifugation at 100***g*** for 3 min at 4°C, the supernatant was removed and the beads were washed six times with 400 µl of BB. The beads were suspended in 2× protein loading buffer, heated at 95°C for 15 min, and 15 µl of each sample was resolved in denaturing SDS polyacrylamide 4–17% gradient gels. In addition, 10% of the input was separated on the same gel.

### Protein techniques

Proteins were separated by SDS-polyacrylamide electrophoresis (SDS–PAGE) using 4–17% gradient gels, and transferred to PVDF membranes (Pierce) according to the manufacturer's protocol. Proteins on the membranes were visualised via PonceauS staining (0.1% w/v, in 5% acetic acid) for 20 min, followed by destaining in 5% acetic acid. Specific proteins were detected using primary antibodies against Gcn1 (1:1000, HL1405, [20]), Gcn2 (1:1000, [61]), Gcn20 (1:1000, CV1317, [20]), eIF2α-P (1:1000, # 44-728G, Invitrogen), Pgk1 (1:5000, # 459250, Invitrogen), myc (1:500, # 11667203001, Roche Applied Science), FLAG (1:500, #F3165, Sigma), and GFP (1:1,000, # sc-8334, Santa Cruz). Immune complexes were then visualised using the Super-signal Chemiluminescence detection substrate (Pierce), and horseradish peroxidase conjugated to donkey anti-rabbit antibodies (#31458, Invitrogen, for the detection of Gcn1, Gcn2, Gcn20, eIF2α-P, and GFP antibodies), conjugated to goat anti-mouse antibodies (#31430, Thermo, for detection of Pgk1 and myc antibodies), conjugated to goat anti-guinea pig antibodies (#A18769, Thermo, for detection of Gcn2), and the LAS4000 chemiluminescence imaging system.

## Open Access

Open access for this article was enabled by the participation of Massey University in an all-inclusive *Read & Publish* agreement with Portland Press and the Biochemical Society under a transformative agreement with CAUL.

## Abbreviations

BB: breaking buffer
GAAC: general amino acid control
Gcn: general control non-derepressible
RWDBD: RWD binding domain
SDS–PAGE: SDS polyacrylamide gel electrophoresis
slg^−^: slow growth
SM: sulfometuron methyl
SM^s^: sulfometuron methyl sensitivity
WT: wild-type
